# The effect of 4-hexylresorcinol on xenograft degradation in a rat calvarial defect model

**DOI:** 10.1186/s40902-016-0076-y

**Published:** 2016-08-05

**Authors:** Yei-Jin Kang, Ji-Eun Noh, Myung-Jin Lee, Weon-Sik Chae, Si Young Lee, Seong-Gon Kim

**Affiliations:** 1 Department of Oral Microbiology, College of Dentistry, Gangneung-Wonju National University, 7 Jukhyun-gil, Gangneung, 25457 Republic of Korea; 2 Gangneung Center, Korea Basic Science Institute, Gangneung, 25457 Republic of Korea; 3 Analysis Research Division, Daegu Center, Korea Basic Science Institute, Daegu, 41566 Republic of Korea

**Keywords:** 4-Hexylresorcinol, Bovine, Bone graft, Degradation

## Abstract

**Background:**

The objective of this study was to evaluate xenograft degradation velocity when treated with 4-hexylresorcinol (4HR).

**Methods:**

The scapula of a cow was purchased from a local grocery, and discs (diameter 8 mm, thickness 1 mm) were prepared by trephine bur. Discs treated with 4HR were used as the experimental group. Untreated discs were used as the control. X-ray diffraction (XRD), Fourier transform infrared spectroscopy (FT-IR), antibacterial test, endotoxin test, and scanning electron microscopy (SEM) were performed on the discs. In vivo degradation was evaluated by the rat calvarial defect model.

**Results:**

The XRD and FT-IR results demonstrated successful incorporation of 4HR into the bovine bone. The experimental disc showed antibacterial properties. The endotoxin test yielded results below the level of endotoxin contamination. In the SEM exam, the surface of the experimental group showed needle-shaped crystal and spreading of RAW264.7 cells. In the animal experiments, the amount of residual graft was significantly smaller in the experimental group compared to the control group (*P* = 0.003).

**Conclusions:**

In this study, 4HR was successfully incorporated into bovine bone, and 4HR-incorporated bovine bone had antibacterial properties. In vivo experiments demonstrated that 4HR-incorporated bovine bone showed more rapid degradation than untreated bovine bone.

## Background

Many types of graft materials have been used for the reconstruction of the maxillofacial region [1]. Although autogenous bone grafting is the gold standard, the amount of available bone is limited, and donor site morbidity has been reported [2]. Xenografts are widely used in the dental field, and many of them are bovine-originated xenografts [3]. For the successful bone graft procedure, timely degradation of the graft is essential for new bone regeneration. However, most xenografts and allografts have poor biodegradability [4, 5]. If the graft is not degraded in a timely manner, the space occupied by the graft cannot be replaced by newly regenerated bone.

4-Hexylresorcinol (4HR) is a family of resorcinolic lipids [6]. 4HR is a well-known antiseptic [7] and reduces the melanosis of food [8]. Therefore, it has been used for oral gargling [7], for treatment of sore throat [9], or as a food ingredient [7]. Recently, its molecular mechanism was unveiled in a cancer cell study. 4HR suppresses calcium oscillation [10] and the nuclear factor-kB (NF-kB) pathway [11]. 4HR can induce cellular differentiation via increasing the expression of keratin 10 and involucrin [12]. These antiproliferative properties of 4HR have synergistic effects when it is used with conventional anticancer drugs such as cisplatin [13].

As 4HR can suppress the NF-kB pathway [11], it may also influence osteoclast activity. The NF-kB pathway is important in osteoclast activation [14]. If 4HR inhibits the NF-kB pathway during bone regeneration, more bone formation can be anticipated. Actually, hydroxyapatite (HA) coating with 4HR in a dental implant shows better bone formation than just HA coating without 4HR [15]. As osteoclasts are also a type of multinucleated cell, 4HR can suppress formation of multinucleated cells [16]. This action is helpful for the inhibition of the reaction to foreign bodies induced by silk materials and results in better bone formation [16]. Silk is a poorly degradable material when it is implanted in the body [17]. 4HR also accelerates degradation of silk graft materials [16, 17].

Collectively, xenografts treated with 4HR have been shown to suppress the reaction to foreign bodies and to promote rapid degradation of poorly biodegradable materials. The objective of this study was to evaluate xenograft degradation velocity upon treatment with 4HR.

## Methods

### Graft preparation

The scapula of a cow was purchased from a local grocery. Using trephine bur (diameter 8.0 mm), round bone grafts were cut at a thickness of 1.0 mm. The control bone was placed into a conical tube containing a 10 % ethanol solution, and the experimental bone was placed in a 10 % ethanol and 3 % 4HR solution. These tubes were placed on a rotating machine for 24 h. Then, the grafts were placed in the drying oven for 8 h. They were sterilized with ethylene oxide gas and stored at room temperature before usage. The weight of dried grafts was measured and the amount of 4HR incorporation into the experimental bone was between 10 to 15 % by weight.

### X-ray diffraction, Fourier transform infrared absorbance spectra, and endotoxin test

X-ray diffraction (XRD) patterns of the samples were collected in the range of 10 to 60° (2θ) using a diffractometer (PANalytical, X’Pert Pro MPD) with a Cu-Kα (λ = 1.5418 Å) radiation source. Fourier transform infrared (FT-IR) spectrum measurements were carried out using a Vertex 80 (Bruker Optics, Germany) spectrometer coupled with a Hyperion 3000 (Bruker Optics, Germany) microscope equipped with a germanium attenuated total reflectance objective lens (ATR ×20) and a liquid nitrogen-cooled mercury cadmium telluride detector.

An endotoxin test was performed using a commercial kit, and the subsequent procedure was in accord with the manufacturer’s protocol.

### Antibacterial test

Two oral pathogens (*Streptococcus sanguinis*, ATCC 10556, and *Aggregatibacter actinomycetemcomitans*, ATCC 33384) and *Staphylococcus aureus* (ATCC 502A) were used for antibacterial tests. The bacterial strains were cultured with brain heart infusion (BHI) (Becton, Dickinson and Company, Sparks, MD, USA) broth under aerobic conditions supplemented with 5 % CO_2_. *S. aureus* was also cultured under aerobic conditions.

The control disc and experimental disc were placed on the surface of blood agar plates (Hangang, Gunpo-si, Korea) for *S. sanguinis* and *S. aureus* and BHI plates for *A. actinomycetemcomitans*. For comparison, two different types of antibiotic discs (vancomycin and penicillin) were also used. The plates of *S. sanguinis* and *A. actinomycetemcomitans* were incubated at 37 °C aerobically supplemented with 5 % CO_2_ for 2 days. For *S. aureus*, the plates were incubated at 37 °C aerobically for 1 day. The maximum diameter of the inhibition zone was observed.

### Scanning electron microscopic examination with EDX microanalysis and cellular attachment assay

The specimens were coated with 0.7 nm of OsO_4_ (HPC-1SW, Japan). Each specimen was observed using a scanning electron microscope (Hitachi, SU-70) and underwent EDX microanalysis (EDAX Genesis; Pv 77, EDAX, USA) to analyze the elements of the area of interest. The compositions of the elements were compared.

Murine macrophages from the Cell Bank (RAW264.7; Korean Cell Line Bank No. 40071) were grown on the control and experimental discs. The growth of the cell culture was stopped at 1 h after seeding by fixing the samples. All materials including raw materials were prepared for scanning electron microscopic examination. After immobilization of the samples on the plate, each sample was coated with gold and examined using a scanning electron microscope (H-800, Hitachi, Japan).

### Animal experiment

This animal study was approved by IACUC (GWNU-2014-14). Seventeen 12-week-old Sprague-Dawley rats with an average weight of 300 g were used for this experiment. A dental-trephine bur was used under copious saline solution irrigation to form a full-thickness calvarial defect. An 8-mm-diameter defect was created on the rat parietal bone, and the control graft or experimental graft was placed in the defect. The control group consisted of eight animals, and the experimental group included nine animals. After the grafting procedure, the pericranium and skin were sutured with 3-0 black silk. The animals were sacrificed 6 weeks after the operation.

### Histological analysis

The calvarial samples were subjected to dehydration and embedding. The segments were embedded to show the sagittal sections in paraffin blocks. The paraffin blocks were sliced (5 μm) and stained with hematoxylin and eosin. The detailed staining procedure followed the standard method in the manufacturer’s manual. The selected sections were photographed with a digital camera (DP-73; Olympus, Tokyo, Japan). The images were analyzed with Sigma Scan Pro (SPSS, Chicago, IL). The ratio of the remaining graft was calculated based on the ratio of the remaining graft to the original size of the graft.

### Statistical analysis

An independent sample *t* test was used to compare the control and experimental groups in the animal study. The significance level was set at *P* < 0.05.

## Results

### 4HR-incorporated bovine bone had antibacterial properties

**Fig. 1:**
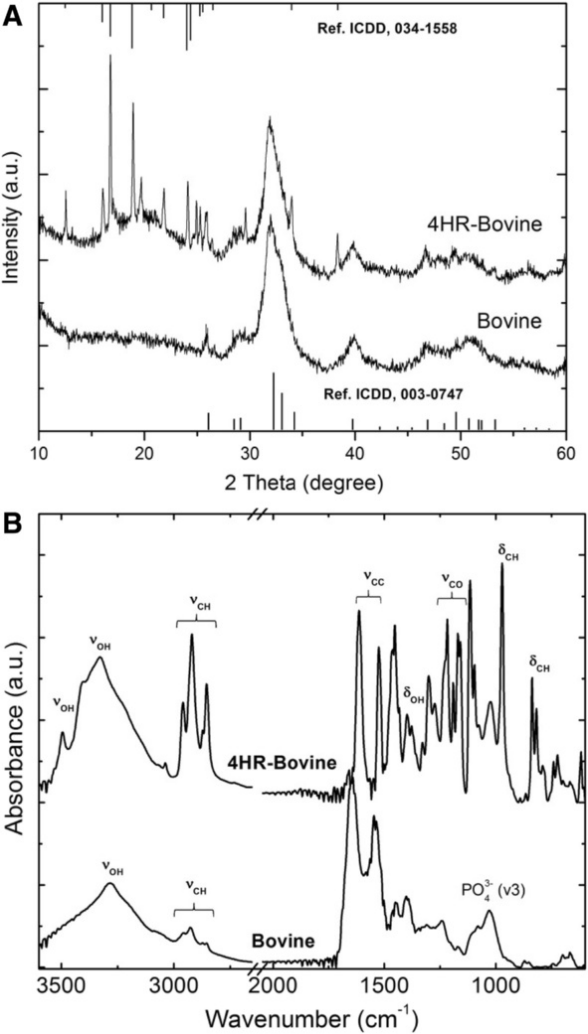
**a** XRD patterns of the bovine and 4-hexylresorcinol (4-HR)-treated bovine samples. **b** FT-IR spectra

Figure 1a shows the XRD patterns of the bovine and 4HR-treated bovine samples. The bovine specimenpresents a typical diffraction pattern corresponding to an HA phase (International Center for Diffraction Data (ICDD), 003-0747), consisting of calcium hydroxide phosphate components. The observed diffraction peaks were somewhat broadened, indicating low crystallinity. 4HR treatment does not change any diffractions of the bovine. Otherwise, additional narrow diffraction peaks were observed in the relatively low angle region, and the pattern was reasonably matched to the reference diffraction of 4HR (ICDD, 034-1558). The narrow diffraction peaks imply high crystalline characteristics.

As seen in the FT-IR spectra, the bovine sample shows several vibrational absorptions: an OH vibration at 3250 cm^−1^ and a PO_4_^3−^ vibration at 1026 cm^−1^ (Fig. 1b). The characteristic vibrations of aliphatic hydrocarbons at 2800 ~ 3000 cm^−1^ and the vibrational absorptions at 1200 ~ 1700 cm^−1^ are attributed to organic moieties in the bovine samples. The 4HR-treated bovine sample clearly shows distinctive infrared absorptions corresponding to 4HR [18, 19]. The OH and aliphatic hydrocarbon peaks are also strengthened in the region of 2800 to 3600 cm^−1^. The 1614 and 1525 cm^−1^ peaks are attributed to aromatic ring C-C stretching. The 1217, 1192, and 1171 cm^−1^ absorptions are attributed to C-O stretching. The vibrational absorption at 972 cm^−1^ is attributed to CH_2_ wagging, and the several absorption peaks at 780 ~ 840 cm^−1^ can be assigned to aromatic C-H bending [19]. A subsequent endotoxin assay yielded results of 0.04 EU/ml for the control disc and 0.01 EU/ml for the experimental disc. These values were below the level of endotoxin contamination (<0.3 EU/ml).

**Fig. 2:**
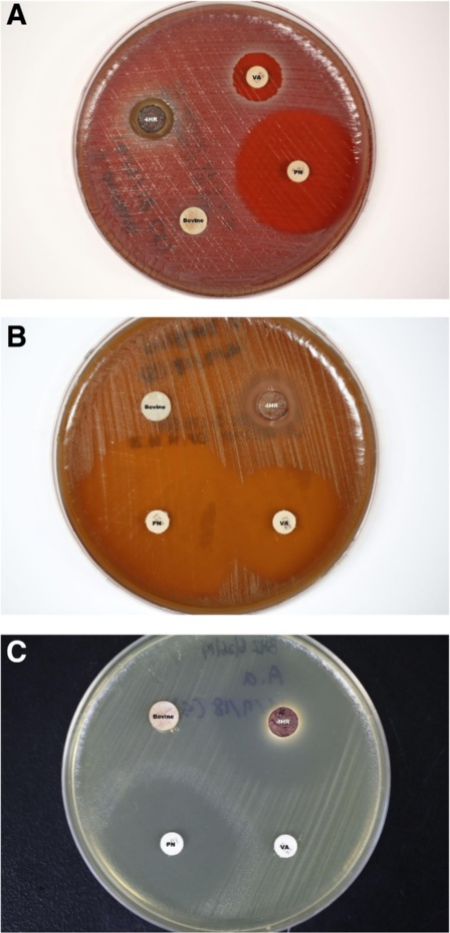
Antibacterial test. **a** *S. sanguinis*, **b** *S. aureus*, and **c** *A. actinomycetemcomitans* (*PN* penicillin, *VA* vancomycin, *4HR* 4-hexylresorcinol-incorporated bovine bone). The 4-hexylresorcinol-incorporated bovine bone disc inhibited the growth of all tested bacteria. Interestingly, vancomycin could not inhibit the growth of *A. actinomycetemcomitans*, while the 4-hexylresorcinol-incorporated bovine bone disc showed a similar inhibition level to the penicillin disc (**c**)

The experimental disc showed a clear bacterial inhibitory zone (Fig. 2). However, the control disc did not have any inhibitory zones. The experimental disc inhibited the growth of all tested bacteria. Interestingly, vancomycin could not inhibit the growth of *A. actinomycetemcomitans*, while the experimental group showed similar inhibition levels for the penicillin disc.

**Fig. 3:**
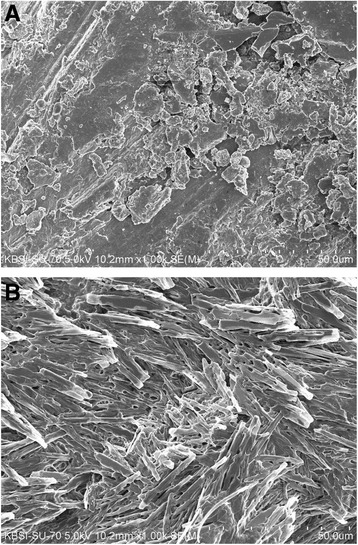
SEM analysis of the graft. The surface of the bovine bone was generally flat with hydroxyapatite crystal and organic material (**a**). However, the surface of the 4-hexylresorcinol-incorporated bovine bone showed multiple needle-like crystals (**b**)

**Fig. 4:**
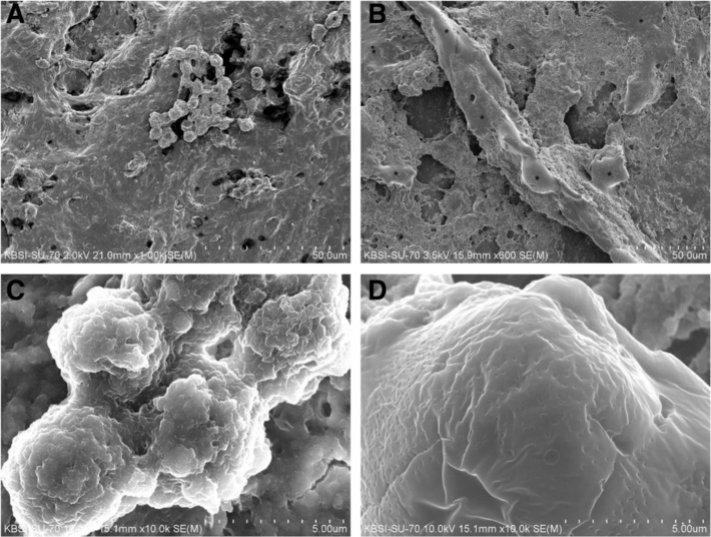
Cellular attachment test. Small, round cells (*asterisk*) were sporadically attached on the surface of bovine bone (**a**). Cellular spreading (*asterisk*) was frequently observed, and multiple round projections on the cellular surface were noticed in the 4-hexylresorcinol-incorporated bovine bone group (**b**). High magnification view demonstrated small cells conglomerated each other in the bovine bone group (**c**). Large cells with multiple round projections were found in the 4-hexylresorcinol-incorporated bovine bone group

In the SEM analysis, the experimental disc showed needle-like crystals on its surface (Fig. 3). Resorcinol is a needle-like crystal that becomes pink upon exposure to light and air [20]. EDX microanalysis demonstrated that the composition of carbon was increased compared to the untreated control (Table 1). The calcium and phosphate contents were also decreased after the 4HR treatment. RAW264.7 cells on the control disc were spherical and did not spread at 1 h after seeding (Fig. 4). However, RAW264.7 cells were well spread on the 4HR-treated bovine disc. Interestingly, numerous submicron-sized processes were observed on the surface of the macrophage.

### Rapid degradation of 4HR-incorporated bovine bone graft

**Fig. 5:**
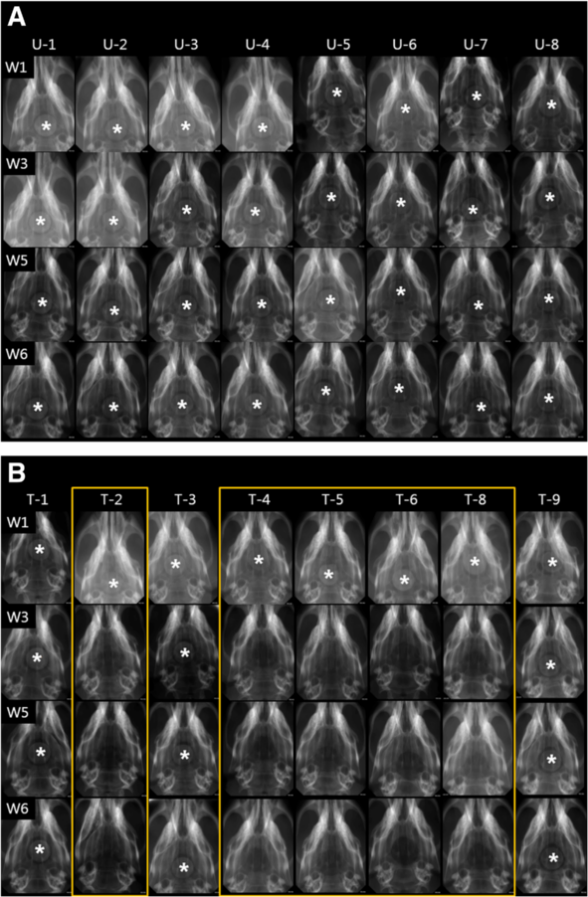
Serial radiograph. The radiograph was taken 1 (W1), 3 (W3), 5 (W5), and 6 (W6) weeks after operation. **a** The animals received bovine bone implants (*asterisk*). All implants were present until 6 weeks after operation. **b** The animals received 4-hexylresorcinol-incorporated bovine bone implants (*asterisk*). Five implants were not observed from 3 weeks after operation (boxed by *yellow line*)

**Fig. 6:**
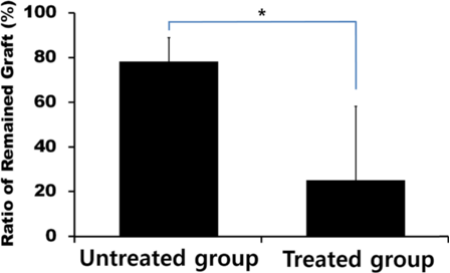
The amount of residual graft was evaluated by histological view. When compared to the untreated group, the 4HR-treated group showed significantly lower residual grafting (*P* = 0.003)

Serial radiography demonstrated rapid degradation in the experimental group (Fig. 5). Six of the nine grafts in the experimental group showed complete degradation at 6 weeks after surgery. The grafts started to disappear at 3 weeks after surgery. However, all grafts were observed in the control group, and there was no significant change in size until 6 weeks. When the amount of residual graft was compared, the difference between the groups was statistically significant (Fig. 6; *P* = 0.003). Two animals that received 4HR-incorporated grafts showed heavy seroma formation on the grafted site. One was drained spontaneously, but the other was observed until 6 weeks after surgery.

**Fig. 7:**
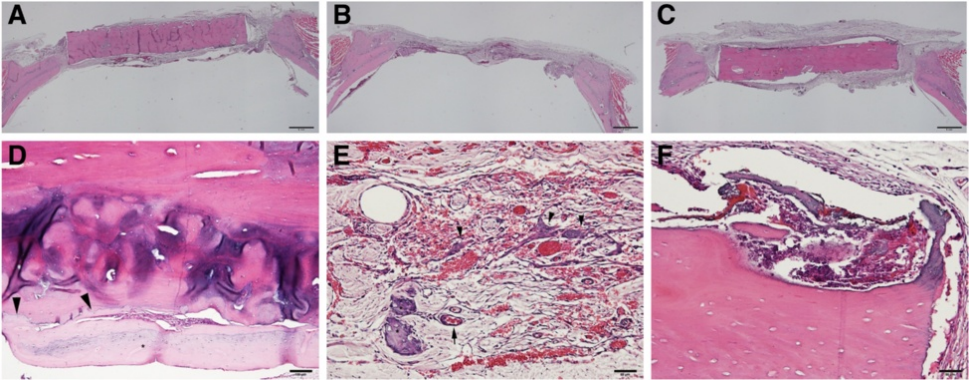
Histological exam. **a** The bovine bone implant was encapsulated by a fibrotic capsule, and no sign of degradation was observed. **b** The animal that received 4-hexylresorcinol-incorporated bovine bone implants showed little residual grafting and still experienced a large bony defect. New bone formation was observed only in the cutting edge of the calvarial bone. **c** Another animal bovine bone implant incorporated with 4-hexylresorcinol showed focal degradation of the graft. Most grafts still occupied the bony defect. **d** New bone regeneration (*asterisk*) over the bovine implant was observed in some animals that received a bovine bone implant. The regenerated bone directly contacted with the implant (*arrow heads*). **e** High magnification view of (**b**). Residual graft was stained with violet color (*asterisk*), and multinucleated giant cells (*arrow heads*) were observed. Highly developed vascular channels were also observed. Interestingly, some giant cells appeared to transform into endothelial cells (arrow head). **f** High magnification view of (**c**). Focal dissolution of implants with reparative calcification was observed

Histological exams are shown in Fig. 7. The untreated control was encapsulated by fibrotic tissue (Fig. 7a). The sample of the complete degradation of the graft demonstrated an almost complete disappearance of the implant with a well-developed vascular channel in the bone defect (Fig. 7b). Some samples from the 4HR-treated group showed minimal degradation of the implant (Fig. 7c). Interestingly, some samples from the untreated groups showed that the newly regenerated bone was in contact with the graft (Fig. 7d). In the experimental group, there were a few regenerated bones in the defect (Fig. 7e). Loose fibrotic tissue occupied the area of grafting. Under high magnification, we observed that multinucleated cells encompassed the residual graft (Fig. 7e). There was a rich vascular network around the residual graft. There were few multinucleated cells. The samples with less degradation showed no difference in gross view compared to those in the control group. Under high magnification, local resorption with reparative bone regeneration was observed (Fig. 7f). However, multinucleated cells were not observed.

## Discussion

In this study, bovine bone obtained from a local grocery was used for the repair of rat calvarial defect after simple processing. 4HR-treated bovine bone had antibacterial properties, and RAW264.7 cells were well attached to the surface of the 4HR-treated bovine bone. 4HR-treated bovine bone had a higher degradation rate than the untreated control. However, its degradation velocity was too rapid and did not contribute to new bone regeneration.

4HR is a family of hydroxyl phenols [6]. 4HR has two –OH groups. A H^+^ ion can be released from –OH groups of the benzene ring, and the 4HR has acidic properties [20]. Therefore, the concentration of 4HR in the bone graft should be carefully monitored. If the concentration of 4HR is too high and is used for HA-based bone graft materials, H^+^ ions released from the graft would decrease the pH locally and result in massive loss of calcium from the graft. Although all prepared graft materials came from the same conical tube, some grafts might not have been exposed to 4HR. As a result, there might be a variation in the 4HR concentration among grafts. Based on the weight change of the graft, the 4HR concentration of each graft was expected to range from 10 to 15 % by weight. HA incorporating gluconic acid is almost completely degraded 2 weeks after surgery [4]. The amount of acid and the release pattern would influence the overall degradation velocity of HA [4]. In a previous study, 3 % wt 4HR in HA or silk was not shown to accelerate graft loss [16]. Therefore, lower concentration of 4HR should be used for bovine bone graft in future study.

4HR can induce cellular apoptosis dependent on its concentration. In the case of epithelial cancer, the apoptosis-inducing concentration is much lower than that of the normal dermal fibroblast [12]. As the 4HR concentrations of grafts were relatively higher than those of a previous study [16], high concentrations of 4HR might induce apoptosis responsible for graft degradation in some cases. Sintered bovine bone graft and synthetic HA are biodegraded by osteoclasts [21] or macrophages [22]. The exact concentration of 4HR required for apoptosis in these cells is yet to be determined. The bovine bone used for the implant may have been treated by heat. However, chemically treated bovine bone has better mechanical properties than heat-treated bone [23]. As 4HR treatment is a chemical treatment, it would not influence the bone’s original mechanical property.

In this study, the control group showed new bone deposition bordering the graft (Fig. 7d). Mixed bovine bone grafted in the rat calvarial defect was not completely degraded at 9 months, while new bone formation was observed in the defect border [24]. There was no inflammation around the graft in the control group (Fig. 7a, d). As the control group was only treated with 24 h ethanol washing, using a graft material process that originates from bovine bone may be considered in the future. The graft materials in this study were sterilized by ethylene oxide. As demonstrated in the EDX analysis results, many organic components may remain in the bovine bone. Bio-Oss also has residual proteins [25]. The ethylene oxide sterilization does not reduce osteoinductivity of bone morphogenetic proteins [26]. Therefore, bone morphogenetic proteins in the bovine bone would not be destroyed by ethylene oxide. Only the concentration of 4HR in the graft appeared to be unknown in this study. The optimal 4HR concentration for bone graft should be determined in future studies.

## Conclusions

In this study, 4HR was successfully incorporated into bovine bone, and 4HR-incorporated bovine bone had antibacterial property. The in vivo experiment demonstrated that 4HR-incorporated bovine bone showed more rapid degradation than untreated bovine bone. However, 4HR-incorporated bovine bone graft did not show elevated new bone formation.
